# Supplementary material for: Enforcement of Fairness Norms by Punishment: A Comparison of Gains and Losses
Source: Behav Sci (Basel). 2024 Jan 5;14(1):39. doi: 10.3390/bs14010039 (PMC10812817; doi:10.3390/bs14010039)
Supplement: Supplementary file 1 [file behavsci-14-00039-s001.zip › Supplementary Material Instructions S2. Game Instructions.pdf]

Enforcement of fairness norms by punishment: A comparison between gains and losses.

Supplementary material S2. Game Instructions.

Experimental Instructions – First sub-session loss treatment

Welcome to this experiment and thank you for your participation!

Procedure of the experiment.
The experiment will be moderated through this conference room. Please keep the window to this room open until the end of the experiment so that we can communicate with you. The experiment itself takes place via a special software that you also open in your browser. You will receive a link for this at the beginning. No software will be installed on your computer! You will receive a questionnaire, which you please fill out carefully.

Rules of conduct.
Basically, we ask you to concentrate on the experiment during the whole experiment. It would be nice if you do not communicate with other people during the experiment and please use your computer only for participating in the experiment! If you have any question, please ask it via the private chat function in the conference room.

Payment.
This is the first of two appointments in this experiment. You will receive a payment of €15.00 today. This can be reduced again on the second appointment. For this you will receive a short questionnaire at the end, in which you commit yourself to participate in the second appointment of the experiment. If anything prevents you from keeping the second appointment, you have to repay the payment minus €3.00 compensation for today's appearance. The payment will be made by bank transfer, for this you will be asked for your account data (name, IBAN, bank) at the very end. This data will be used exclusively for the payout and will be deleted after the transfer!

Experimental Instructions – Second sub-session loss treatment

General instruction

Welcome to this experiment and thank you for your participation!

Procedure of the experiment.
The experiment will be moderated through this conference room. Please keep the window to this room open until the end of the experiment so that we can communicate with you. The experiment itself will take place via a special software that you also open in your browser. You will receive a link for this at the beginning. No software will be installed on your computer!

The experiment consists of a game for losses. This starts with instructions. Please read them carefully! Afterwards you will receive two questionnaires. You and all other participants make your decisions independently of the other participants.

Rules of conduct.
Basically, we ask you to concentrate on the experiment during the whole experiment. It would be nice if you do not communicate with other people during the experiment and please use your computer only for participating in the experiment (any distraction could influence your decisions, we would like to avoid that)! If you have any question, please ask it via the private chat function in the conference room. Please do not ask questions publicly, as this could influence the decisions of the other participants!

Loss and repayment.
You have already received a payout of €15.00 on the first appointment of this experiment. This amount may decrease again during this part of the experiment depending on your own decisions and the decisions of the other players. Your loss will be settled at the end of the experiment. The repayment will be made by bank transfer. You will receive an invoice for this.

Instruction dictator game with third-party punishment – losses.

Game description

The game consists of two stages. Participants are randomly divided into groups of three players each (Player 1 / Player 2 / Player 3).

Stage 1

Player 1 shares a loss of €-10.00 with player 2.

Player 1:

Player 1 receives a loss of €-10.00. He is to divide this between himself and Player 2.

Player 1 chooses his own share of the loss on a number line (in 50 cent increments).

Player 2:

Player 2 receives the difference, thus €-10 less than the share of Player 1.

Player 1 decides independently of Player 2. Player 2 makes no decision in this game.

Stage 2

Player 3 decides whether to penalize Player 1.

Player 3:

Player 3 is presented with the split between Player 1 and Player 2 and decides whether to punish Player 1. He has the choice between

> Penalize Player 1: Player 1 is assigned an additional loss of €-2.00 [weak: €-1.00, strong: €-6.00]. Player 3 is assigned a loss of €-2.00 for this.
>
> Do not punish: Player 1 bears the share of the loss according to the distribution from stage 1. Player 3 is not credited with any loss.

Player 2's share of the loss does not change in stage 2.

Screen-shots Dictator game with third-party punishment: loss treatment.

Decision page: player 1.

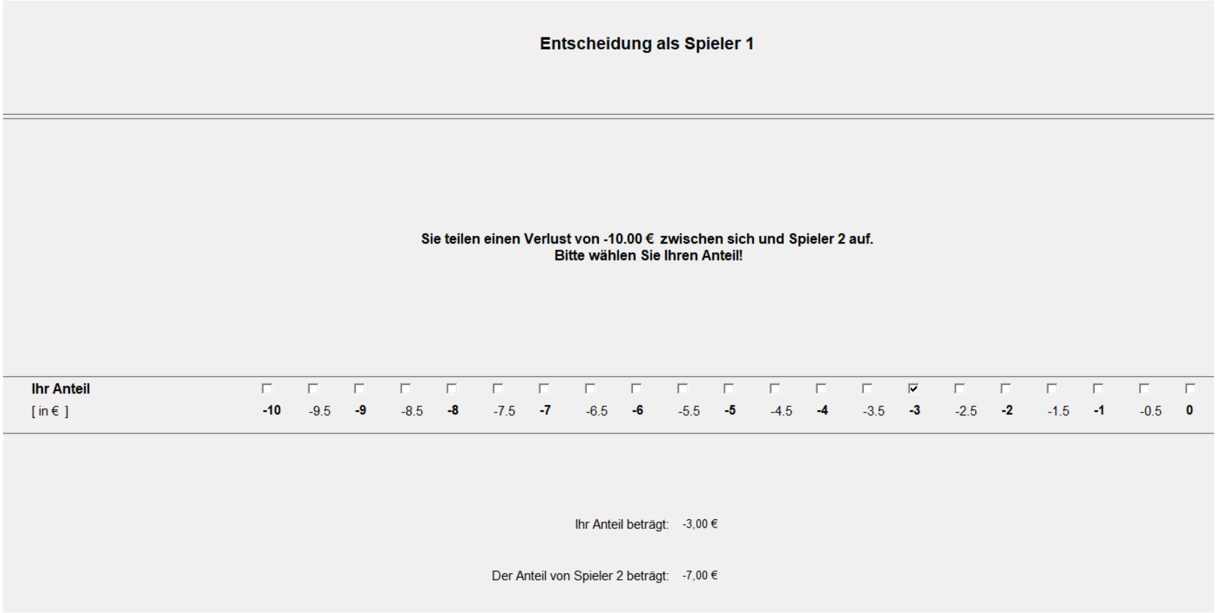

Decision page: player 3.

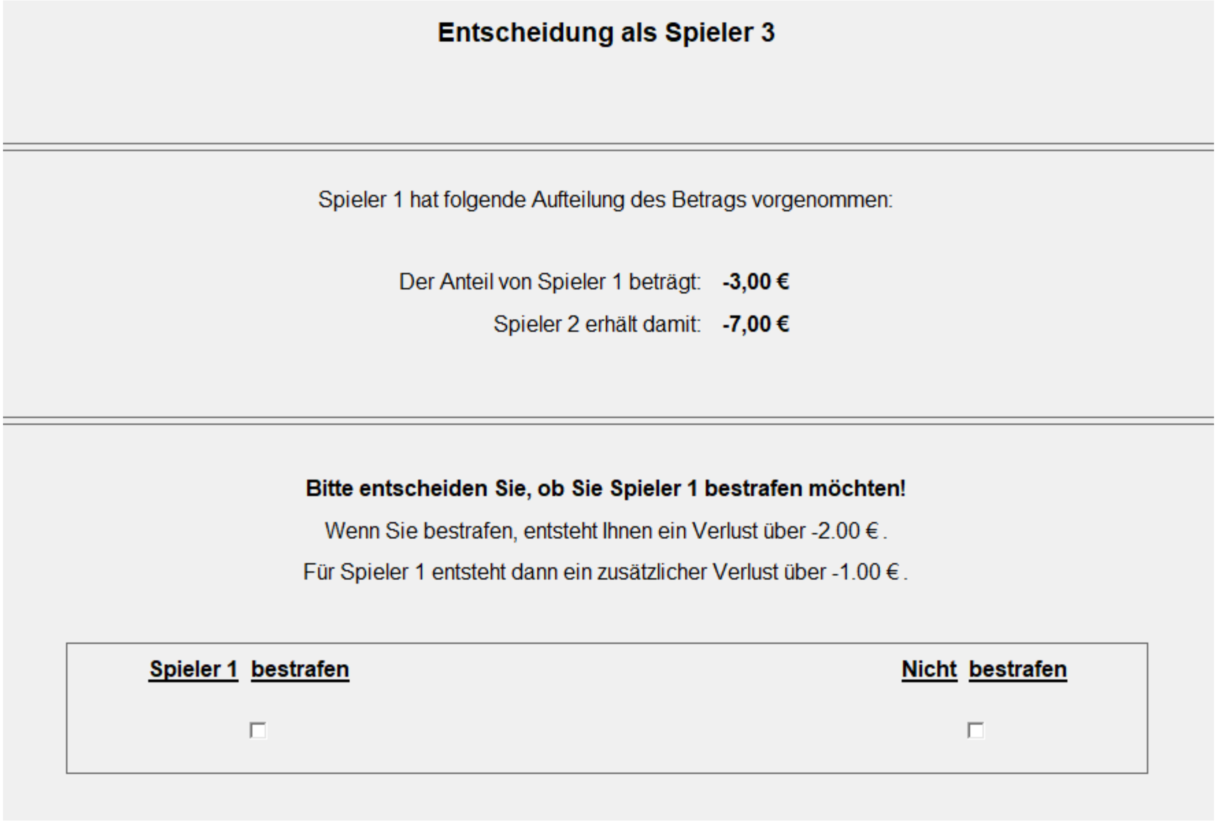

Experimental Instructions – gains treatment

General instruction

Welcome to this experiment and thank you for your participation!

Procedure of the experiment.

The experiment will be moderated through this conference room. Please keep the window to this room open until the end of the experiment so that we can communicate with you. The experiment itself will take place via a special software that you also open in your browser. You will receive a link for this at the beginning. No software will be installed on your computer! The experiment consists of a game. This starts with instructions. Please read them carefully! Afterwards you will receive some questionnaires. You and all other participants make your decisions independently from the other participants.

Rules of conduct.

Basically, we ask you to concentrate on the experiment during the whole experiment. It would be nice if you do not communicate with other people during the experiment and please use your computer only for participating in the experiment (any distraction could influence your decisions, we would like to avoid that)! If you have any question, please ask it via the private chat function in the conference room. Please do not ask questions publicly, as this could influence the decisions of the other participants!

Winning and payment.

For your participation you will already receive a prize of €5.00. You can increase this payout in the following depending on your own decisions and the decisions of the other participants. Your winnings will be settled at the end of the experiment. The payout will be made by bank transfer, for this you will be asked for your account data (name, IBAN, bank) at the very end. This data will only be used for the payout and will be deleted after the transfer!

Instruction dictator game with third-party punishment – gains.

Game description

The game consists of two stages. Participants are randomly divided into groups of three players each (Player 1 / Player 2 / Player 3).

Stage 1

Player 1 splits an amount of €10.00 with player 2.

<u>Player 1</u>:

Player 1 receives an amount of €10.00 to divide between himself and Player 2.

Player 1 chooses his own share of the gain on a number line (in 50 cent increments).

<u>Player 2</u>:

Player 2 receives the difference, thus €10.00 less than the share of Player 1.

Player 1 decides independently of Player 2. Player 2 makes no decision in this game.

Stage 2

Player 3 decides whether to penalize Player 1.

<u>Player 3</u>:

Player 3 is credited with an amount of €10.00.

Player 3 is presented with the split between Player 1 and Player 2 and decides whether to punish Player 1. He has the choice between

> <u>Penalize Player</u> 1: Player 1 will have €2.00 [weak: €1.00, strong: €6.00] deducted from his winnings. Player 3 will have €2.00 deducted from his winnings for this.
>
> <u>Do not punish</u>: Player 1 receives a payout according to the distribution from stage 1. Nothing is deducted from Player 3's winnings.

The payout for Player 2 does not change in stage 2.

Screen-shots Dictator game with third-party punishment: gains treatment.

Decision page: player 1.

Entscheidung als Spieler 1

Sie teilen einen Betrag von 10.00 € zwischen sich und Spieler 2 auf.
Bitte wählen Sie Ihren Anteil!

**Ihr Anteil** [in €]	☐ **0**	☐ 0.5	☐ **1**	☐ 1.5	☐ **2**	☐ 2.5	☐ **3**	☐ 3.5	☐ **4**	☐ 4.5	☐ **5**	☐ 5.5	☐ **6**	☐ 6.5	☐ **7**	☐ 7.5	☐ **8**	☑ 8.5	☐ **9**	☐ 9.5	☐ **10**

Ihr Anteil beträgt: 8,50 €

Der Anteil von Spieler 2 beträgt: 1,50 €

Decision page: player 3.

Entscheidung als Spieler 3

Spieler 1 hat folgende Aufteilung des Betrags vorgenommen:

Der Anteil von Spieler 1 beträgt: **8,50 €**

Spieler 2 erhält damit: **1,50 €**

Bitte entscheiden Sie, ob Sie Spieler 1 bestrafen möchten!

Wenn Sie bestrafen, entstehen Ihnen hierdurch Kosten über 2.00 € .

Bei der Auszahlung von Spieler 1 werden dann 2.00 € abgezogen.

Spieler 1 bestrafen	Nicht bestrafen
☐	☐
